# Identification of histamine receptor subtypes in skeletal myogenesis

**DOI:** 10.3892/mmr.2014.3073

**Published:** 2014-12-10

**Authors:** YAN CHEN, VASILY STEGAEV, VESA-PETTERI KOURI, TARVO SILLAT, PAUL L. CHAZOT, HOLGER STARK, JIAN GUO WEN, YRJÖ T. KONTTINEN

**Affiliations:** 1 Department of Medicine, Institute of Clinical Medicine, University of Helsinki, Biomedicum 1, Helsinki 00029, Finland; 2 Department of Anatomy, University of Helsinki, Biomedicum 1, Helsinki 00029, Finland; 3 Department of Urology, Pediatric Urodynamic Center, Institute of Clinical Medicine, The First Affiliated Hospital of Zhengzhou University, Zhengzhou, Henan 450052, P.R. China; 4 School of Biological and Biomedical Sciences, Durham University, Durham DH1 3LE, UK; 5 Institute of Pharmaceutical Chemistry, Goethe University, Frankfurt D-60438, Germany; 6 ORTON Orthopedic Hospital of the ORTON Foundation, Helsinki 00280, Finland; 7 COXA Hospital for Joint Replacement, Tampere 33520, Finland

**Keywords:** myogenesis, differentiation, histamine receptor type 3, histamine

## Abstract

To date, conventional and/or novel histamine receptors (HRs) have not been investigated in mouse skeletal myogenesis. Therefore, the present study aimed to investigate the HR-subtypes in skeletal myogenesis. The myogenesis of C2C12 skeletal myoblasts was evaluated using desmin, myogenin and myosin heavy chain (Myh) as early, intermediate and late differentiation markers, respectively. Reverse transcription-quantitative polymerase chain reaction and immunostaining were performed and the messenger RNA (mRNA) expression levels of the HR-subtypes and markers were determined. H_1_R mRNA was found to be highly expressed in myoblasts at day 0; however, the expression levels were reduced as differentiation progressed. By contrast, H_2_R mRNA expression remained constant, while H_3_R mRNA expression increased by 28-, 103- and 198-fold at days 2, 4 and 6 compared with the baseline level (day 0), respectively. In addition, Myh expression increased by 7,718-, 94,487- and 286,288-fold on days 2, 4 and 6 compared with the baseline expression level (day 0). Weak positive staining of the cells for H_3_R protein was observed on day 2, whereas highly positive staining was observed on days 4 and 6. HR expression during myogenesis was, in part, regulated by the stage of differentiation. These results along with previous findings indicated possible involvement of H_1_R in the regulation of progenitor cell mitogenesis and of H_2_R in the relaxation of acetylcholine-stimulated contraction of muscle cells, following the activation of professional histamine-producing cells, including mast cells. By contrast, H_3_R may participate in the regulation of specialized myocyte functions, potentially by maintaining the relaxed state under the influence of constitutive H_3_R activity and low histamine concentrations, locally produced/released by non-professional histamine-producing cells.

## Introduction

Histamine is a well-known biogenic and cationic amine, which is synthesized, stored and released by professional histamine-synthesizing cells. Mast cells, basophils and enterochromaffin cells contain the endoplasmic 54 kDa histidine decarboxylase (HDC), which converts L-histidine to histamine (1). Histamine is released into and stored within storage granules, prior to regulated release (1). Following activation of professional histamine-producing cells, a burst release results in a transient high histamine concentration in the extracellular space. These transient histamine concentrations are sufficient to stimulate the conventional histamine receptors, histamine receptor type 1 [H_1_R; binding affinity (pK_i_) = 4.2] and histamine receptor type 2 (H_2_R; pK_i_ = 4.3) (2). Smooth muscle cells (3,4), cardiomyocytes (5,6) and skeletal muscle tissue (7) express these conventional histamine receptors, which regulate cellular proliferation and the contraction state of the cells stimulated via the histamine/H_1_R or H_2_R axes (3–6).

A previous study identified that the cytoplasmic 73 kDa ‘pro-form’ of HDC produced histamine, however, at a 100–1,000-fold lower rate compared with the typical enzyme isoform of the professional histamine-synthesizing cells (1). In non-professional histamine-producing cells, histamine is released into the cellular cytoplasm rather than being stored, and is therefore not subjected to regulated burst release (8). These cells contain organic cation transporters, which are equilibrative uniporters and transport the intracellularly synthesized histamine from the non-professional histamine synthesizing cells along the histamine concentration gradient to the extracellular space (8). Histamine concentrations achieved in this manner are not sufficient to stimulate conventional histamine receptors. Therefore, this mechanism was hypothesized to represent an ancestral vestigium of a function that had become obsolete during phylogenesis. However, studies conducted within the last decade that focus on G-protein coupled receptors have revealed novel members of the histamine receptor family (2). These novel histamine receptors, histamine receptor type 3 (H_3_R; pK_i_ = 8.0) and histamine receptor type 4 (H_4_R; pK_i_ = 8.2), have >10,000-fold greater affinity for histamine compared with the conventional receptors (2). In addition, the low basal levels of histamine produced by non-professional histamine-producing cells, including dendritic cells (9) and lymphocytes (10,11), have been demonstrated to be sufficient in order to bind to and regulate cells equipped with these novel, high-affinity histamine receptors. The role of high histamine concentration in the regulation of muscle cell tone was investigated in previous studies (3–6). Studies using histamine receptor agonists and/or antagonists have suggested that novel histamine receptors may also be present and functional in the bronchial smooth muscle cells at least (3). However, to date, no studies indicating the presence of histamine receptors at the messenger RNA (mRNA) and protein level in myoblasts, myocytes or myotubes during skeletal myogenesis have been reported. Due to the presence and role of H_1_R, H_2_R and H_3_R in the function of other muscle cell types, the present study aimed to assess whether striated muscle cells synthesize and express the histamine receptors, H_3_R and H_4_R. In addition, the current study investigated whether these receptors are developmentally regulated during myogenesis in association with various markers of myogenic maturation.

## Materials and methods

### Cell culture

The present study was approved by the institutional Medical Ethics Committee of the Institue of Clinical Medicine, University of Helsinki (Helsinki, Finland) and was performed in accordance with the 1983 Declaration of Helsinki. Mouse C2C12 myoblasts were obtained from the Turku Center for Biotechnology, University of Turku (Turku, Finland) (12), and maintained in growth medium comprising Dulbecco’s modified Eagle’s medium (DMEM; Lonza/BioWhittaker, Walkersville, MD, USA) supplemented with 10% fetal bovine serum (FBS; HyClone, GE Healthcare Life Sciences, Little Chalfont, UK), antibiotics (100 U/ml penicillin and 100 μg/ml streptomycin; Lonza) and 200 mM L-glutamine (Lonza) at 37°C in a humidified 5% CO_2_ atmosphere. The composition of the differentiation medium was similar to the growth medium, with the exception of FBS, which was reduced from 10% to 1%. The cells were passaged using trypsinization (0.5% trypsin in 0.5 mM EDTA; Gibco-BRL Life Technologies, Carlsbad, CA, USA) from the culture plate at 80% confluence.

### Reverse transcription-quantitative polymerase chain reaction (RT-qPCR)

To investigate the expression of histamine receptors in C2C12 myogenesis, 50,000 cells/well were seeded in 12-well plates (CellStar; Greiner Bio-One, Frickenhausen, Germany). The cells were initially grown in growth medium for two days to reach 80% confluence. Next, the medium was exchanged with differentiation medium to induce myogenesis. Total RNA was isolated from the cells at days 0, 2, 4 and 6 using an RNeasy Mini kit (Qiagen, Düsseldorf, Germany) according to the manufacturer’s instructions. Total RNA (1 μg) was reverse transcribed using iScript cDNA Synthesis kit (Bio-Rad Laboratories, Inc., Hercules, CA, USA). RT-qPCR was performed with 100 ng first-strand cDNA using iQ SYBR^®^ Green Supermix (Bio-Rad Laboratories, Inc.) in an iCycler iQ5 Multicolor Real-Time PCR Detection system (Bio-Rad Laboratories, Inc.). Primers for mouse desmin (Des), myogenin (Myog), myosin heavy chain IIa (Myh2), H_1_R, H_2_R, H_3_R, H_4_R and porphobilinogen deaminase (PBGD) genes were designed using the National Center for Biotechnology Information Primer-Blast tool (Table I; http://www.ncbi.nlm.nih.gov/tools/primer-blast/; accessed: 01/03/2012). The mRNA copy numbers of the samples analyzed were determined in triplicate and normalized against the PBGD gene.

### Immunofluorescence staining

The C2C12 cells were seeded at 2×10^4^ cells/well in 24-well plates (CellStar) on coverslips and grown in growth medium for two days to reach 80% confluence, followed by culturing in differentiation medium to induce myogenesis. Differentiated cells from days 0, 2, 4 and 6 were fixed for 15–20 min in 4% paraformaldehyde (Sigma-Aldrich, St. Louis, MO, USA) with phosphate-buffered saline (PBS; 10mM phosphate buffer, 140 mM saline; pH 7.4), washed three times in PBS (5 min each time) and in 0.5% Triton X-100 (Thermo Fisher Scientific, Fair Lawn, NJ, USA)/PBS for 15 min to permeabilize the cells. Subsequently, the cells were cultured under the following conditions sequentially: i) 10% normal donkey serum (Jackson Immunoresearch Laboratories, Inc., West Grove, PA, USA) for 1 h; ii) 1 μg/ml polyclonal peptide affinity purified rabbit anti-human desmin (1:200), myogenin (1:400) or myosin heavy chain (Myh) immunoglobulin G (IgG; 1:400) antibodies (obtained from Dr John E. Erikson, University of Turku, Turku, Finland) (12), or rabbit anti-human H_3_R polyclonal antibodies (1:1,000; LS-A476; MBL International, Woburn, MA, USA) at 4°C overnight and washed three times in PBS (5 min each time). Non-immune rabbit IgG (1:1,000; 1 μg/ml; R&D Systems, Minneapolis, MN, USA), was used at the same concentration as the primary antibodies as a negative staining control; iii) secondary antibody AlexaFluor^®^488-conjugated monoclonal donkey anti-rabbit IgG (1:400; Invitrogen Life Technologies, Carlsbad, CA, USA) in 0.1% bovine serum albumin (Sigma-Aldrich)-PBS for 1 h and washed three times in PBS (5 min each time); iv) DAPI dye (Sigma-Aldrich; 1:2,000 in distilled water) for 5 min. The coverslips were washed twice in PBS and distilled water for 10 min, prior to mounting with Vectashield medium (Vector Laboratories, Inc., Burlingame, CA, USA). Labeled slides were analyzed and photographed using a Leica DM 6000 B/M fluorescence microscope, with a motorized Leica XY-stage connected to a Leica DFC 420 digital camera, and analyzed using the Leica Application Suite Advanced Fluorescence 2.5.0.6735 software (Leica Microsystems GmbH, Wetzlar, Germany).

### Statistical analysis

SPSS software, version 17.0 (SPSS, Inc., Chicago, IL, USA) was used to perform statistical analyses in addition to Matlab (MathWorks, Inc., Natick, MA, USA), which was used to perform the Mann-Whitney U test. All values are presented as the mean ± standard error of the mean. P<0.05 was considered to indicate a statistically significant difference between values.

## Results

### Myogenesis of C2C12 cells

**Figure 1:**
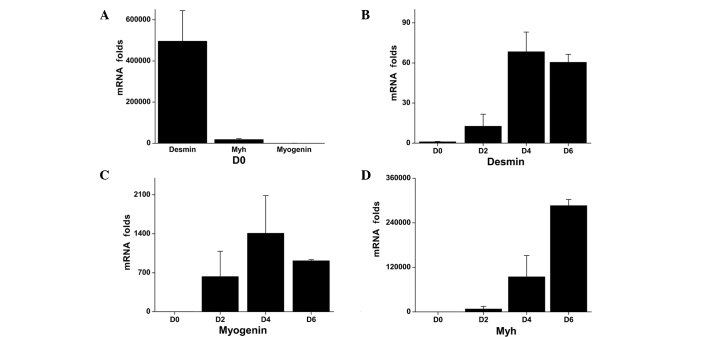
Myogenesis differentiation marker expression levels, determined by reverse transcription-quantitative polymerase chain reaction. (A) Relative expression levels of myogenic differentiation markers desmin (early marker), myogenin (intermediate marker) and Myh (late marker) in C2C12 cells at day 0, and relative expression level variations in (B) desmin, (C) myogenin and (D) Myh during myogenesis at days 2, 4 and 6. The data are expressed as the mean ± standard deviation. Myh, myosin heavy chain; mRNA, messenger RNA; D, day.

RT-qPCR was used to detect the mRNA expression levels of the early, intermediate and late myogenesis markers, desmin, myogenin and Myh2, respectively, during differentiation. On day 0, desmin was expressed in myoblasts at significantly higher levels compared with the myogenin or Myh (Fig. 1A). The desmin expression levels increased during myogenesis, reaching 12-, 68- and 60-fold over the baseline level (day 0), on days 2, 4 and 6, respectively (Fig. 1B). On day 0, the myogenin mRNA exxpression levels were low; however, the mRNA expression levels increased by 631-, 1,408- and 914-fold at days 2, 4 and 6, respectively (Fig. 1C). Desmin and myogenin expression levels peaked on day 4, whereas the expression of Myh, a late myogenesis marker, continued to increase over the entire study period, reaching 7,718-, 94,487- and 286,288-fold higher expression levels at days 2, 4 and 6, respectively, compared with the baseline level (Fig. 1D).

**Figure 2:**
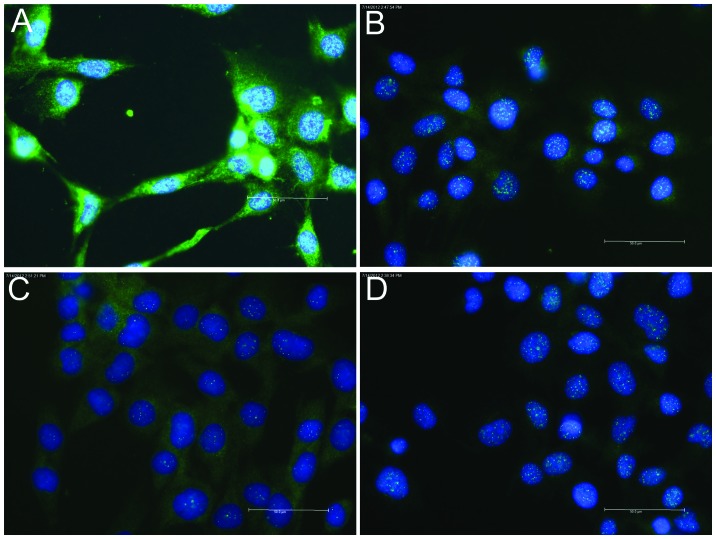
Immunofluorescent staining (green) of (A) desmin, (B) myogenin, (C) histamine receptor type 3 and (D) negative control at day 0 in undifferentiated C2C12 cells. Staining of the late myogenic marker myogenin heavy chain was negative (not shown). DAPI was used for nuclear counterstaining (blue color). Scale bar, 50 μm.

**Figure 3:**
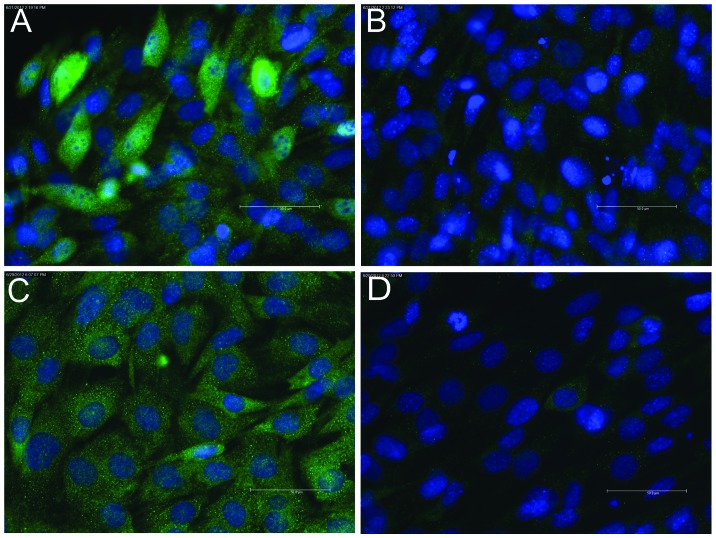
Immunofluorescent staining (green) of (A) myogenin, (B) myosin heavy chain, (C) histamine receptor type 3 and (D) negative control at day 2 in differentiated C2C12 cells. DAPI was used for nuclear counterstaining (blue color). Scale bar, 50 μm.

**Figure 4:**
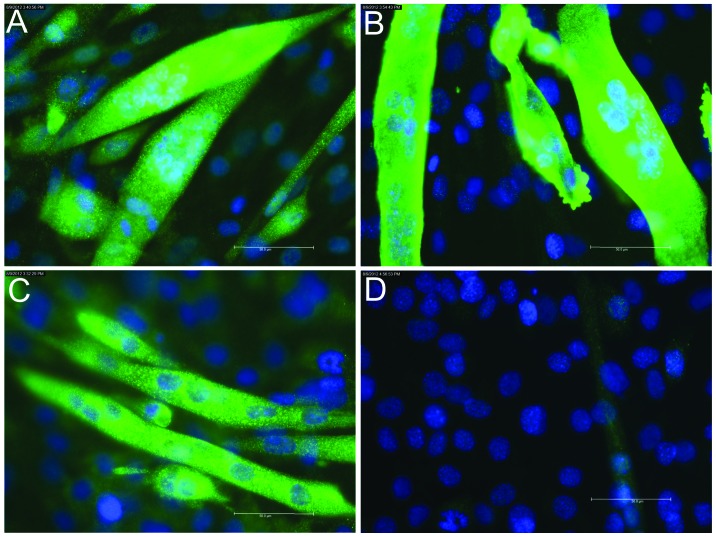
Immunofluorescent staining (green) of (A) myogenin, (B) myosin heavy chain, (C) histamine receptor type 3 and (D) negative control at day 6 in differentiated C2C12 cells. DAPI was used as a nuclear counterstain (blue color). Scale bar, 50 μm.

Indirect immunofluorescence staining of the myogenesis marker proteins revealed positive staining of the early marker, desmin, at day 0 (Fig. 2A); however, no staining was observed for the intermediate marker, myogenin (Fig. 2B), or the late marker, Myh (data not shown). On day 2, staining for myogenin was found to be positive (Fig. 3A), whereas staining for Myh remained negative (Fig. 3B). On days 4 (data not shown) and 6, positive staining for myogenin (Fig. 4A) and Myh (Fig. 4B) was detected.

### Expression of histamine receptors

**Figure 5:**
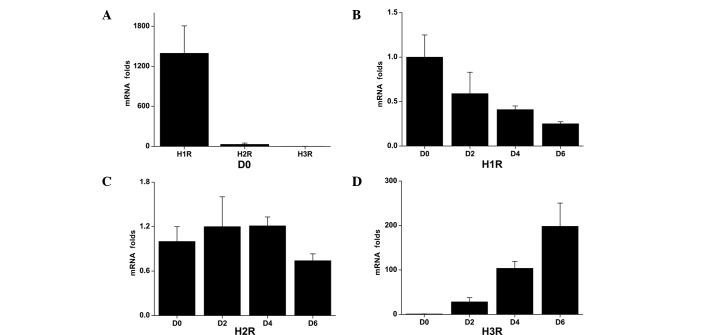
Histamine receptor subtype expression levels, determined by reverse transcription-quantitative polymerase chain reaction. (A) Relative expression levels of H_1_R, H_2_R and H_3_R in C2C12 cells at day 0, and relative expression level variations of (B) H_1_R, (C) H_2_R and (D) H_3_R during myogenesis (days 0–6). The data are expressed as the mean ± standard deviation. mRNA, messenger RNA; H_n_R, histamine receptor type n (where n=1, 2 or 3).

RT-qPCR was used to detect the mRNA expression levels of histamine receptors associated with the differentiation stages (Fig. 5). H_1_R mRNA was found to be highly expressed in C2C12 myoblasts (day 0), whereas expression was decreased during the differentiation process (Fig. 5A and B). By day 6, the expression level decreased to ~25% of the baseline level (day 0). H_2_R mRNA was also expressed in C2C12 cells and the expression levels remained relatively constant throughout the differentiation process (Fig. 5A and C). The expression of H_3_R was found to be low in C2C12 myoblasts; however, following differentiation, the expression levels increased by 28-, 103- and 198-fold over the baseline level on days 2, 4 and 6, respectively (Fig. 5A and D). H_4_R mRNA expression was not detected at any time-point.

Indirect immunofluorescence staining for H_3_R protein during the myogenesis of C2C12 cells revealed almost negative staining at day 0 (Fig. 2C), weakly positive staining on day 2 (Fig 3C) and strongly positive staining on days 4 (data not shown) and 6 (Fig. 4C).

## Discussion

To the best of our knowledge, the present study demonstrated for the first time that striated muscle cells expressed H_1_R, H_2_R and H_3_R-coding mRNA and corresponding receptor proteins, but lacked receptor, H_4_R. The lack of H_4_R in striated muscle cells may be due to the fact that H_4_R(+) cells have been previously been identified in the bone marrow, thymus and spleen, as well as at the cellular level in bone marrow-derived cells, including mast cells, basophils, eosinophils, neutrophils, dendritic cells and lymphocytes (13).

Investigation of the early, intermediate and late phases of myogenesis was performed using desmin, myogenin and Myh as markers, respectively. The results indicated that histamine receptors were dynamically regulated during differentiation, suggesting that they may have distinct regulatory functions. H_1_R presented the highest expression in myoblasts on day 0, compared with the other receptors; however, the expression levels of H_1_R were subsequently decreased during myogenesis. H_2_R expression was found to be low on day 0 and remained relatively constant throughout all the phases of myogenesis. By contrast, H_3_R showed the lowest expression in myoblasts on day 0; however, the H_3_R expression levels were subsequently increased, and continued to increase throughout myogenesis.

The low affinity of H_1_Rs for histamine requires burst release from professional histamine-synthesizing cells in order to induce target cell effects. Notably, in cardiomyocyte precursor cells, H_1_Rs are abundant and regulate Ca^2+^ oscillation and frequency. In such progenitor cells, this process is coupled with the entry of cells into the cell cycle and bromodeoxyuridine incorporation (5). The results of the present study, which revealed high levels of H_1_R expression during early myogenesis, along with the aforementioned previous observations, suggested that high histamine levels may stimulate myoblast proliferation during the early phases of differentiation. This hypothesis is further supported by the observations of a previous study, which demonstrated that mast cell precursors migrated from bone marrow to skeletal muscle tissue in 17 to 20-day-old rat fetuses, indicating interactions between the professional histamine-producing mast cells and skeletal muscle cells in proliferation or differentiation (14).

In the present study, H_2_R expression remained constant throughout all the phases of myogenesis, and thus, may be involved in the maintenance of relaxation following burst release of histamine (since H_2_R stimulation requires high histamine concentrations), with a curare-like effect (which is a competitive antagonist of the nicotinic acetylcholine receptor) (15). By contrast, H_2_R antagonists have been demonstrated to possess an anti-cholinesterase activity (16).

Due to the high affinity of H_3_R for histamine, the non-professional histamine-producing cells are able to stimulate H_3_R-expressing cells. The levels of histamine released by the non-professional histamine-producing cells are not sufficient to activate the conventional, low-affinity receptors (2). Furthermore, in contrast to the conventional H_1_R and H_2_R, H_3_R has a relatively high constitutive activity level, which is ~25% active in the absence of H_3_R-ligands (17,18). According to the two-state model of receptor activation, G-protein coupled receptors exist in equilibrium between an active and inactive receptor state. Upon ligand binding, the G-protein becomes activated (R^*^) and begins to ‘couple’ and transduce the extracellular stimulus into an intracellular signal, while ligand-free G-protein coupled receptors exist in a passive, uncoupled conformation. However, H_3_R spontaneously acquires the R^*^ state, which promotes G-protein-mediated signaling in the absence of an agonist. Therefore, H_3_R is hypothesized to have significant constitutive functions in mature myocytes and myotubes, which are independent of burst release (cellular emergencies) and driven by the low histamine concentrations generated by non-professional histamine-producing cells and by their constitutive activity (17,18).

High histamine concentrations are known to mediate the pathological contraction of smooth muscles cells in the bronchiolar walls, including during acute attacks of asthma and anaphylactic reactions mediated by H_1_R. H_1_R is coupled to Gα_q/11_ protein, which cleaves phosphatidylinositol 4,5-bisphosphate to diacylglycerol and inositol 1,4,5-trisphosphate, via the activation of phospholipase C. This results in Ca^2+^ influx and initiates smooth muscle contraction (3). Notably, low histamine concentrations act as potent relaxant agents for pre-contracted smooth muscle cells via H_3_Rs (3). In the present study, the time course of H_3_R expression during myogenesis indicated that H_3_R may have long-term, constitutive effects on mature skeletal muscles cells, rather than being activated under exceptional circumstances that results in burst release of the histamine stores from mast cells and basophils. Based on the findings of Cardell and Edvinsson (3) and the long-term low histamine level-induced and constitutive H_3_R function, H_3_R was hypothesized to maintain the relaxed state of mature skeletal muscle cells.

In conclusion, further studies are required in order to determine the functions and potential signalling pathways by which the expression of the three histamine receptor subtypes, examined in the present study, are regulated during myogenesis in skeletal muscle cells. Future research may elucidate novel information regarding the etiology and potential treatment of skeletal muscle diseases.

## Figures and Tables

**Table I:** Primer sequences used in reverse transcription-quantitative polymerase chain reaction and the corresponding amplicon lengths.

Gene	Forward primer	Reverse primer	Length (bp)
Des	5′-GCCCTCAAGGGCACCAACGA-3′	5′-TTGCTCGGGGCTGGTTTCTCG-3′	297
Myog	5′-CCCAACCAGCGGCTGCCTAA-3′	5′-GTAGGGTCAGCCGCGAGCAA-3′	245
Myh2	5′-AGCTGCACCTTCTCGTTTGCCA-3′	5′-CGGTCAGGGTCGCTCCTGCT-3′	261
H_1_R	5′-CACTGGAGGCTGCCCTTGTGC-3′	5′-CACCAGCAGGTTGAGGCCCAC-3′	167
H_2_R	5′-TCCTAAGCGACCCGGTACAGC-3′	5′-ATGGAGACTGAGGCACTGCTGG-3′	208
H_3_R	5′-TTCGAGCCTCCGCACCCAGAA-3′	5′-GGTCCAACGGCCGGTCAGC-3′	118
H_4_R	5′-TGCTCAGGTCCCCTTGGCATTT-3′	5′-ACGTGAGGGATGTACAGAGGAATGG-3′	189
PBGD	5′-AAAGTGCCGTGGGAACCAGC-3′	5′-CAGCCACAGCCAGGACGATG-3′	156

Des, desmin; Myog, myogenin; Myh2, myosin heavy chain IIa; H_n_R, histamine receptor type n (n=1–4); PBGD, porphobilinogen deaminase.
